# Concomitant Gastrointestinal Stromal Tumor of the Stomach and Gastric Adenocarcinoma in a Patient with Billroth 2 Resection

**DOI:** 10.1155/2013/583856

**Published:** 2013-12-22

**Authors:** Federico Sista, Valentina Abruzzese, Mario Schietroma, Gianfranco Amicucci

**Affiliations:** Dipartimento di Scienze Cliniche Applicate e Biotecnologie, Università degli Studi di L'Aquila, 67100 Coppito, Italy

## Abstract

*Background*. With this study we focus on the etiopathogenesis and on the therapy of the simultaneous occurrence of Gastric gastrointestinal stromal tumor (gGIST) and adenocarcinoma of the stomach in a patient with Billroth II gastric resection (BIIGR). We report the first case of this event and a review of the literature. *Methods*. A 70-year-old man with a BIIGR, affected by adenocarcinoma of the stomach, was successfully treated with total gastrectomy. The histological examination showed a gastric adenocarcinoma with a synchronous GIST sized 2 cm and S-100, CD117, and CD34 positive. The mutation of PDGFR gene was detected. *Discussion*. This tumor is a rare mesenchymal neoplasm of the gastrointestinal tract. Few cases of synchronous gastric adenocarcinoma and GIST are observed in the literature and no case in patients with BIIGR. Various hypotheses have been proposed to explain this occurrence. It is frequently attributed to Metallothioneins genes mutations or embryological abnormalities, but this has not been proven yet. We suggest a hypothesis about the etiopathogenesis of this event in a BIIGR patient. *Conclusion*. GIST may occur synchronously with gastric adenocarcinoma. This simultaneous occurrence needs more studies to be proven. The study of Cajal cells' proliferation signalling is crucial to demonstrate our hypotesis.

## 1. Introduction

gGISTs are rare; they represent 0.2% of all gastrointestinal tumors [1]. They are also the most common mesenchymal tumors of the gastrointestinal tract. Inappropriately classified in the past as Leiomyomas, Leiomyoblastomas, and Schwannomas, in the late 1990s they were classified as a single neoplastic entity, when immunohistochemistry had identified the gene KIT (CD117) [2]. They derive from Cajal cells arising from cells of the smooth muscle of the viscera [3, 4]. 50–60% of GIST are localized in the stomach, 30–40% in the small intestine, and 5–10% in the colon and rectum. GISTs represent 1% of gastric malignant tumors; they are symptomatic in 60% of cases although the diagnosis is often incidental [4, 5]. Unlike gastric carcinomas, that are often associated with other cancers such as lymphomas, GISTs are rarely synchronous to other gastric neoplasms. Here we report a rare case of gGIST synchronous to adenocarcinoma in a patient with a previous Billroth II gastric resection (BIIGR).

## 2. Case Report

**Figure 1:**
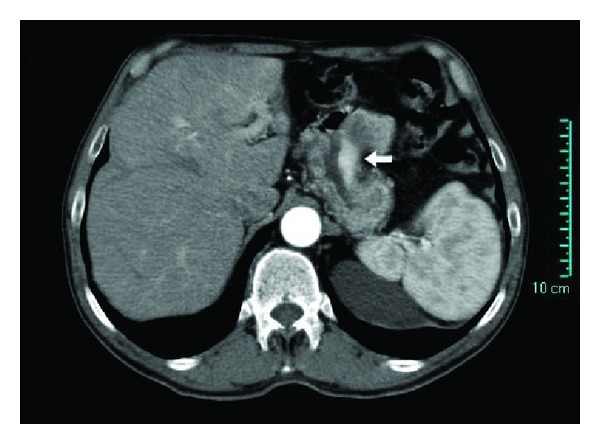
Computer tomography with intravenous contrast of the abdomen showing a thickening of gastric stump (white arrow).

**Figure 2:**
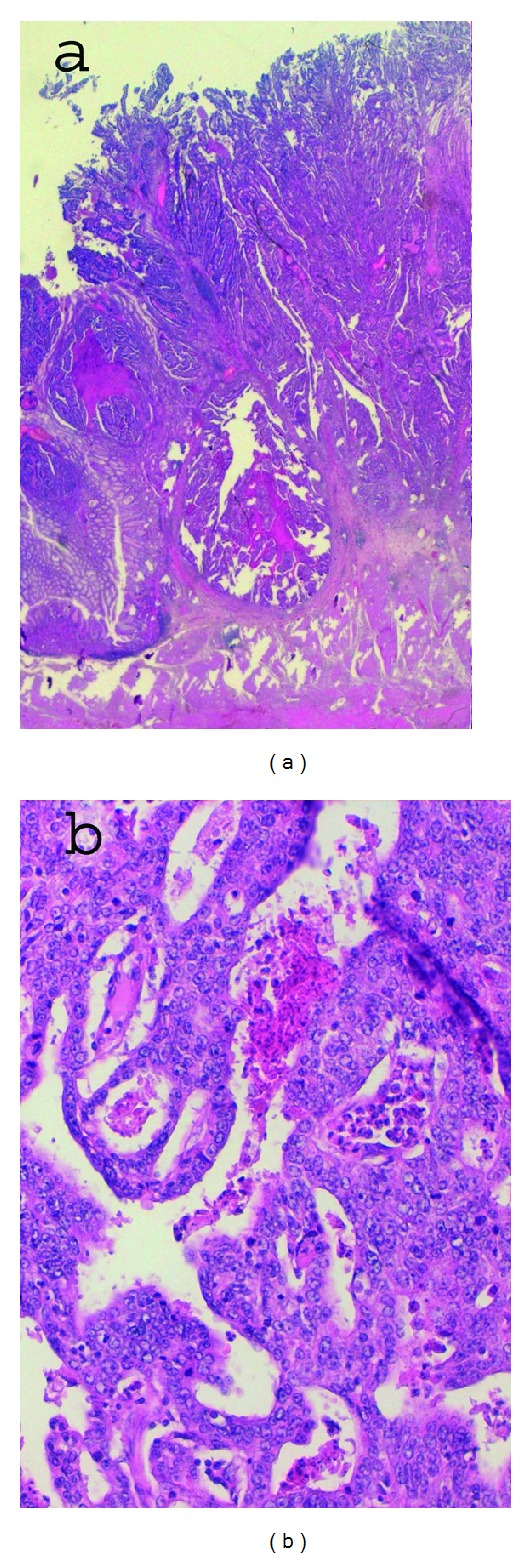
Gastric adenocarcinoma: (a) adenocarcinoma infiltrating depth muscular wall—10x and (b) moderately differentiated tubulo-papillary adenocarcinoma—20x.

**Figure 3:**
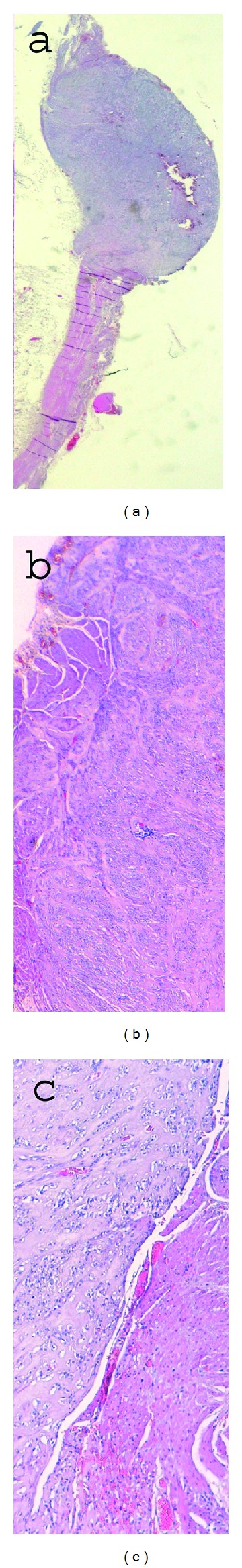
coexistent GIST EE: (a) GIST of the muscle wall of the gastric corpus—2x, (b) fusiform low grade GIST cells composed of spindle cells with ovoid nuclei arranged in short fascicles (nuclear palisading) —4x and (c) interface between GIST and gastric muscle wall—10x.

**Figure 4:**
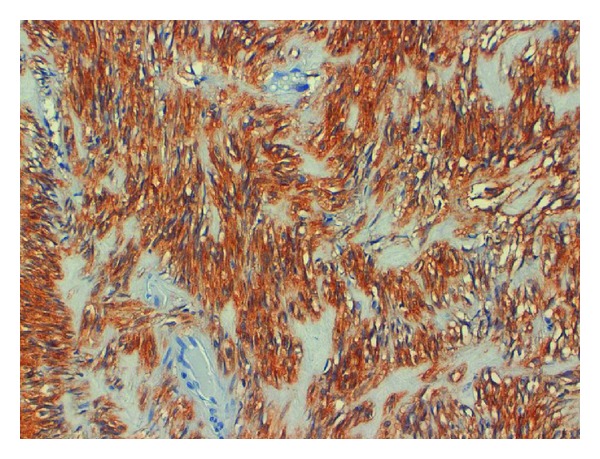
Immunohistochemical features of GIST: c-KIT/CD117 (tyrosine kinase growth factor receptor) positivity—20x.

**Figure 5:**
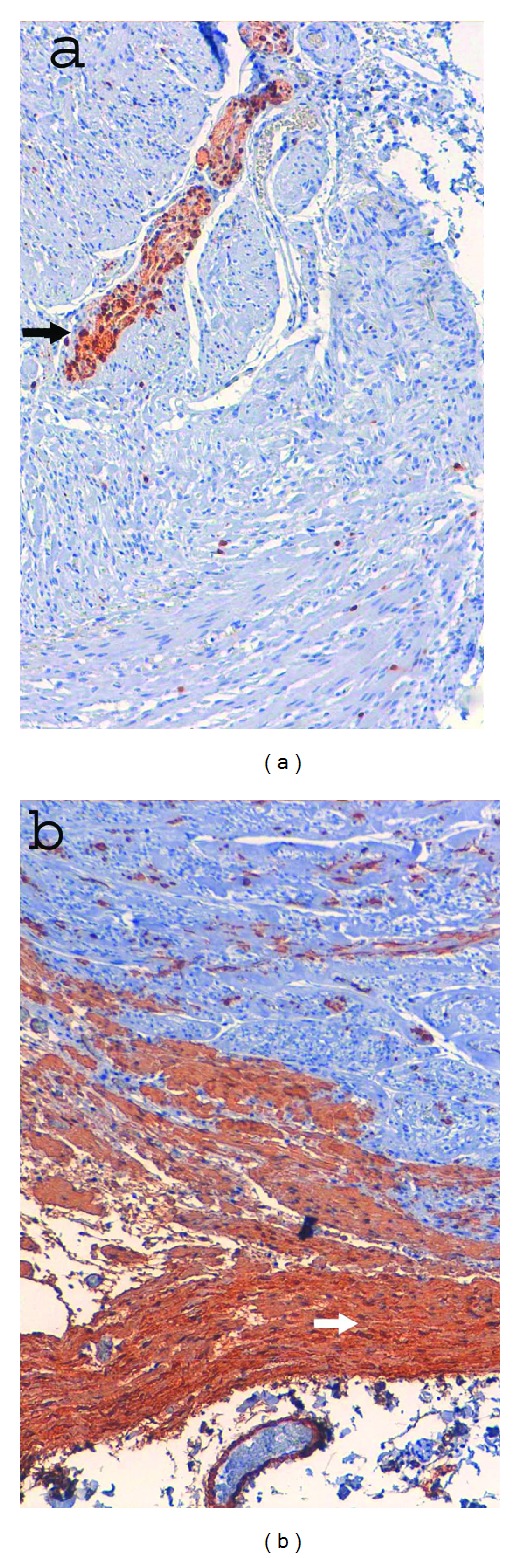
Immunohistochemical features of GIST: (a) S-100 negativity. Instead overexpressed in nerve cells (black arrow) —10x (b) SMA (smooth muscle actin) negativity. Instead overexpressed in muscular gastric wall cells (white arrow) —10x.

A 70-year-old man suffered from dyspepsia, chronic anemia, and epigastric pain for about six months, and he also had a weight loss of 15 kg and melena. The patient underwent BIIGR for a gastric ulcer 35 years before. A gastroscopy was performed, and it showed a “polypoid neoformation that occupies one third of the anastomosis and extends along the greater curvature of the gastric stump for about 3 cm.” Biopsies were performed, and they showed “adenocarcinoma infiltrating the stroma and focally the smooth muscle.” Bioumoral findings (CEA, GICA, AFP, and NSE) were negative. The abdominal computed tomography (CT), executed for the staging of the neoplasia, showed parietal thickening of the gastric stump without secondary repetitions (Figure 1). The patient then underwent a total gastrectomy with a Roux-en-Y reconstruction and D2 lymphadenectomy. The histological examination of the surgical specimen showed a “moderately differentiated tubulo-papillary adenocarcinoma infiltrating deep into the muscle layer” (Figure 2). A 3 cm GIST of the gastric corpus muscle layer coexists. It is composed by proliferating spindle-shaped cells with nuclear palisading (Figure 3). Its cellularity is low, its mitotic index (number of mitoses per 50 high-power fields) is 1/50 HPF, and scattered hyalinization and calcification are observed. There was positivity for KIT/CD117 (Figure 4) and negativity for SMA (smooth muscle actin) and S-100 (Figure 5). The molecular analysis of this tumor showed a PDGFR gene mutation; this error makes the tumor sensitive to the oral treatment with Imatinib Mesylate (Glivec @). The final staging was T3 N2 M0. The postoperative was normal without complications. The patient was discharged in the IXth postoperative day and entrusted to an oncologist consultant.

## 3. Discussion

GIST is a rare cancer that originates from Cajal cells, interstitial pacemaker cells [1, 3, 4]; it shows positivity to tumor markers that can be found also in normal Cajal cells [1, 3]. The immunohistochemical examination highlights the over expression of KIT or CD34 gene in 90% of cases. Therefore, the positivity for the KIT gene is enough to make anatomopathological diagnosis of GIST [2, 3, 6]. Other histological features are the negativity to S-100 and SMA (smooth muscle actin), over expressed in nerve cells and muscular cells (Figure 5), respectively; in this way it is possible to make differential diagnosis with gastric Schwannoma and Sarcoma. Genetic analysis of the PDGFRA is necessary to evaluate an eventual postoperative therapy with Imatinib Mesylate. Over expression of PGFRA is not associated with its mutation; it occurs in up to 85% of cases, while the mutation occurs in up to 5–7% [6–8].

Just few cases in the literature report GISTs synchronous to gastric adenocarcinomas [9–14]. In our case there was a low grade GIST (according to the Bucher Grading System [15]), associated with an infiltrating adenocarcinoma. The percentage of loco regional lymph node repetitions is estimated from 1 to 3,4% [16–18] for GISTs (in our case it was 0%: the repetitions found derived from the adenocarcinoma). Disease progression and patients' outcome depend on tumor's size and on mitosis number [15]. Patients with malignant gGIST have usually a better survival if compared to those with an intestinal localization. In fact, patients with malignant gGIST who undergo a complete resection have a recurrence rate of about 50%, a median time to recurrence of 18–24 months, and a 5-year survival of 50% [8]. Liver and peritoneum are the main organs involved in case of relapse [8, 19]. Liver is involved in more than 60% of cases, representing the only repetition site in 44% of cases [8], while extra-abdominal repetitions are found just in the advanced phases of the disease. Novitsky et al. [20] reported a 92% of 5-year disease-free survival for 4, 4 cm tumors. Other authors reported similar results for gGISTs up to 5 cm [21]. Chaudhry and DeMatteo [8] highlighted that the responsivity to the biological treatment with Imatinib determines a better survival, which is 100% in the first 2 year, with a disease progression just in 61% of cases. The refractoriness to this biological treatment results in a 2-years survival of 36%, with a disease progression in 100% of cases. In our case, the disease-free survival was 18 months for the metastatic progression of gastric adenocarcinoma, without the biological therapy.

GIST in combination with other synchronous malignant disease is a rare event. The studies reported in the literature about this tumor are still few. Some authors [14, 22, 23] report that about 20% of patients with GIST develop another type of tumor, but they do not suggest a common etiology. Liu et al. [14] carried out a study of 54 GISTs synchronous to other digestive tract malignant neoplasia; they showed that the highest incidence of synchrony occurs with esophagus squamous cell carcinoma, with gastric adenocarcinoma and with pancreatic cancer, in a percentage of 1.13%, 0.53% and 0.38% respectively. They also found a higher prevalence in females. In all cases the most frequent location was the gastric corpus. Some other authors noted a higher prevalence of synchrony between GIST and female genital neoplastic pathology. Li et al. reported: “As the full spectra of ovarian epithelial neoplasm may develop in the endometrium and other anatomic components of the female upper genital tract, an “extended or secondary Müllerian system” has been proposed to describe the similarity of the female upper genital tract in common undergoing metaplastic changes giving rise to synchronous neoplasm” [23]. In this respect they hypothesized an embryological origin of these neoplasm. Kawanowa et al. [24] analyzed 100 cases of gastric cancer; they found a high incidence of microscopic GIST (35%), suggesting that other genetic changes (in addition to the gene kit) may be necessary for the evolution in malignant form. These alterations are probably involved even in the genesis of gastric carcinomas. Finally some authors suggested that the coexistence of these neoplasms can be attributed to mutations in Metallothioneins genes; their lack could reduce the cellular oncosuppression necessary to block the onset and progression of GISTs, as well as other epithelial tumors [25, 26].

However, no study highlights if there is a higher GIST preponderance in gastrectomized subjects. In patients with BIIGR in fact, biles' role in the genesis of gastric adenocarcinoma on the anastomotic mucosa is known; there is no evidence so far for an analogous effect for GIST. A common metabolic pathway may be shown through the study of the proliferation signal. It is known that Cajal cell proliferation persists throughout the postnatal period. KIT and Ki-67 signal are observed in the proliferation and development of this kind of cell [27]. Ki-67 protein is associated with cellular proliferation and it can be detected in the cell nucleus during the active phases of the cell cycle [28]. Ki-67 is thus a useful marker to determine the growth fraction of normal or neoplastic cell population. The *Ki-67 labeling index* assesses the fraction of Ki-67-positive tumor cells and is often correlated with the clinical course of cancer [29].

We propose that the genesis of these neoplasms can be the overstimulation of the enteric sympathetic plexus as a result of increased gastric clearance. The absence of a parasympathetic stimulus due to vagotomy can lead to the hypertrophy and hyperplasia of Cajal cells, pacemakers of the enteric sympathetic plexus. The study of Cajal cells' proliferation signal in GISTs and in healthy muscular tunica of resected stomachs could demonstrate the etiopathogenesis of these tumors, justifying the synchronism with adenocarcinomas in gastrectomized subjects. This kind of research could provide useful information on the origins of these tumors, but also they could be the starting point for new therapies. Anyway, because of the GIST's low malignant potential, its probability of recurrence is considerably lower than synchronous gastric adenocarcinoma's one. For this reason, incidental GISTs should not be over treated, in order not to invalidate the outcome of surgical and oncological treatment of the primary disease.

## 4. Conclusion

gGIST in combination with other synchronous malignant diseases is a rare event. GIST's malignancy is considerably lower than gastric adenocarcinoma's one; in this respect, the surgeon has to pay attention to a possible primary GIST, synchronous to other primary malignancies. The coexistence of these neoplasms is an intriguing oncologic model. There is not a unique interpretation of the biochemical mechanisms behind this synchronism, and further studies are needed. Our hypothesis is that in gastrectomized patients there is an overstimulation on Cajal cells, because an increased clearance is necessary. PGFRA mutation affects the treatment of GIST and therefore its prognosis. The GIST overtreatment in terms of survival however could not be effective in case of synchronous neoplasm, because of the worse prognosis of the other tumor. Further studies on the molecular biology of these neoplasms are necessary to detect their biochemical mechanisms; if we could understand the genesis of these synchronisms we also could be able to produce new therapies, such as the biological one.
